# Complete Genome Sequence of Bradyrhizobium ottawaense OO99^T^, an Efficient Nitrogen-Fixing Symbiont of Soybean

**DOI:** 10.1128/MRA.01477-18

**Published:** 2018-11-29

**Authors:** Hai D. T. Nguyen, Sylvie Cloutier, Eden S. P. Bromfield

**Affiliations:** a Ottawa Research and Development Centre, Agriculture and Agri-Food Canada, Ottawa, Ontario, Canada; Georgia Institute of Technology

## Abstract

We present the complete genome sequence of Bradyrhizobium ottawaense strain OO99^T^, a nitrogen-fixing bacterium from root nodules of soybean. The genome consists of a single 8.6-Mb chromosome and includes a symbiosis island. Genes involved in symbiotic nitrogen fixation, stress response, resistance to antibiotics, and toxic compounds were detected.

## Abstract (precis)

We present the complete genome sequence of Bradyrhizobium ottawaense strain OO99^T^, a nitrogen-fixing bacterium from root nodules of soybean. The genome consists of a single 8.6-Mb chromosome and includes a symbiosis island.

## ANNOUNCEMENT

Some species of soil bacteria belonging to the genus Bradyrhizobium have the ability to form nitrogen-fixing symbioses with economically important legumes, including soybean (Glycine max). In previous studies (1, 2), strains of symbiotic bacteria from root nodules of soybean grown in Ottawa, Canada, were characterized and described as the species Bradyrhizobium ottawaense. The type strain of this species, OO99 ( =  LMG 26739^T^  =  HAMBI 3284^T^), is highly efficient in symbiotic nitrogen fixation with soybean (1).

A draft genome sequence of B. ottawaense OO99^T^ was previously obtained with Illumina technology, but the assembly was fragmented (150 contigs; GenBank accession number NPNY00000000) (3). In this study, we resequenced strain OO99^T^ using PacBio technology. Bacteria from a pure culture were grown on yeast extract mannitol agar medium (2). Genomic DNA was extracted using the Promega Wizard SV genomic DNA purification system and cleaned with a Qiagen DNeasy PowerClean Pro kit. Sequencing was done at the Genome Quebec Innovation Centre in Montreal, Canada, with the Pacific Biosciences (PacBio) RS II single-molecule real-time (SMRT) platform (4). A total of 108,802 polymerase reads with an average read length of 13,248 bp were generated; estimated genome coverage was 141-fold. Reads were *de novo* assembled with the hierarchical genome assembly process (HGAP) (5). In the preassembly step, quality control was performed by aligning short subreads on long subreads with BLASR (6); assembly was performed with Celera Assembler (7) and polished with Quiver (5).

The complete genome of Bradyrhizobium ottawaense OO99^T^ comprises a single circular chromosome of 8,606,328 bp with an average G+C content of 63.8%. A total of 8,238 genes, 8,180 coding sequences, 51 tRNAs, and a single rRNA operon were found with the NCBI Prokaryotic Genome Annotation Pipeline v. 4 (PGAP-4) (8, 9). Analyses performed with the PATRIC v. 3.5.26 platform (10) indicated that the most abundant genes were those involved in metabolism (1,084 genes), energy (391 genes), membrane transport (245 genes), and protein processing (238 genes). Genes implicated in motility, chemotaxis, siderophore production, polysaccharide biosynthesis, and stress response (heat, cold, and osmotic shock) were found. We also identified 196 genes involved in resistance to antibiotics and toxic compounds, including arsenic and chromium.

A total of 25 putative genomic islands were detected based on Island Path-DIMOB prediction implemented in the IslandViewer 4 platform (11). A symbiosis island (about 527 kb) flanked by two integrase genes was found. The symbiosis island carries genes required for nodulation and nitrogen fixation. The *nodZ* gene and the regulatory genes *nodD1* and *nodD2* that are implicated in host specificity were present (12). Type I, II, II/IV, and III secretion systems genes and those required for conjugative transfer were also detected.

Further analysis of the OO99^T^ genome will contribute to the knowledge of economically important symbiotic bacteria and will be useful for evolutionary and taxonomic studies of the genus Bradyrhizobium.

### Data availability.

This whole-genome shotgun project has been deposited at DDBJ/ENA/GenBank under the accession number CP029425. Raw PacBio data have been deposited in the NCBI Sequence Read Archive under the BioProject accession number PRJNA470945.
